# Association between the early diagnosis of Crohn’s disease (CD) and the development of its clinical complications among adults at King Abdulaziz University Hospital.

**DOI:** 10.1097/MS9.0000000000003489

**Published:** 2025-07-16

**Authors:** Fatma Khinaifis Al Thoubaity, Rana Mazin Albukhari, Shahad Ghazi AlQurashi, Yasmeen Saeed Alghamdi, Modhi Sultan Mohammed, Nouf Turki Ashgan, Lujain Yousef Nabalawi, Jana Fahad Batarji, Nuha Ibrahim Qashaa, Rahaf Ahmed Alsaedi, Noor Ibraheem Basheikh

**Affiliations:** a Department of General Surgery, King Abdulaziz University Faculty of Medicine, Jeddah, Saudi Arabia; b Medical student, King Abdulaziz University Faculty of Medicine, Jeddah, Saudi Arabia

## Abstract

**Background:**

Despite a steady increase in cases, concerns persist about how early diagnosis affects Crohn’s disease (CD) progression.

**Objectives:**

To assess the association between early (CD) diagnosis and clinical complications among adults.

**Methods:**

A retrospective study was conducted on 149 patients aged 18 years or older who were admitted anddiagnosed with CD between 2000 and 2022. Data on demographics, admission portal, family history, risk factors, past medical history, clinical presentations, complications, recurrence, disease-modifying agents, and laboratory investigations were collected. Diagnostic delay was defined as the time from diagnosis to the 75th percentile.

**Results:**

The mean age was 35.85 ± 12.54 years, with a mean disease duration of 7.72 ± 4.02 years; the average time from symptom onset to diagnosis was 1.93 ± 3.2 years, and from the diagnosis to complications was 2.2 ± 3.0 years. Males comprised 57% of patients, and 80.5% were S nationals. Colonoscopy with biopsy diagnosed 64.4%, and 8.7% had a family history of CD. Of these, 118 (79.2%) had an early diagnosis, while 31 (20.8%) had a late diagnosis. Late-diagnosed patients had a significantly longer time to diagnose complications and were more likely to be non-S. They also had a higher prevalence of kidney oxalate calculi and Asacol use, while early-diagnosed patients had a reduced appetite and fatigue. Multivariate logistic regression found no significant risk factors for late diagnosis.

**Conclusion:**

Delayed CD diagnosis is associated with kidney oxalate calculi. The link between early diagnosis and clinical complications remains inconclusive, requiring further multicenter longitudinal studies.

**HIGHLIGHTS**

- The true incidence and prevalence of inflammatory bowel disease (IBD) in Saudi Arabia remain unknown due to the lack of population-based studies.
- The most common complications noted in patients with a late diagnosis of CD were fistulae, abscesses, and intestinal obstruction, all of which required surgical interventions.
- Colonoscopy with biopsy remains the gold standard for diagnosing Crohn’s disease.
- The long-term outcomes showed that early immunosuppressants or anti-TNF medications notably decreased the risk of CD-related surgeries.

## Introduction

Crohn’s disease (CD) is a chronic inflammatory bowel disease (IBD) involving any segment of the gastrointestinal tract. It is characterized by recurrent episodes of active inflammation interspersed with periods of remission ^[1]^. It affects a substantial portion of the population and has significant social and economic implications ^[2]^.

A retrospective study indicates that the prevalence of CD has been increasing globally, particularly in Western nations, Asia, and the Arab world, with a notable rise in its incidence observed in Saudi Arabia ^[2,3]^. The true incidence and prevalence of IBD in our country remain unknown due to the lack of population-based studies. However, regional retrospective studies have suggested that the incidence of CD and UC is increasing and is consistent with global trends ^[2,4]^.

Recent research suggests that potential risk factors for developing CD include several gene mutations, chronic use of proton pump inhibitors or antibiotics, increased visceral obesity, and smoking ^[5,6,7]^. Although CD can affect individuals of all ages, it commonly manifests in younger populations. Patients with CD frequently present with symptoms such as right lower quadrant pain, bloating, fever, weight loss, and mucoid or bloody diarrhea ^[2,8]^. Definitive diagnosis and suitable treatment may take months or years. For instance, a study in central Saudi Arabia estimated that the duration from the onset of symptoms until the definitive diagnosis was 11 months ^[2]^. Methods of CD diagnosis include clinical signs and symptoms, imaging modalities such as endoscopy with biopsy, and laboratory tests ^[9]^.

Uncontrolled bowel inflammation in CD can lead to long-term complications, including fibrotic strictures, enteric fistulae, and intestinal neoplasia ^[10]^. Chronic intestinal failure is a rare yet serious complication of CD, with many cases requiring permanent dependence on total parenteral nutrition, potentially resulting in bone metabolism disorders ^[11]^.

Treatment options for CD complications include conservative management, percutaneous drainage, and surgical intervention ^[10,11]^. Growing evidence suggests that early intensive therapy with immunomodulators and/or biological agents is associated with a higher likelihood of achieving mucosal healing and sustained remission without the need for steroids ^[12]^. Although CD has no cure, treatment and lifestyle modifications can help maintain remission and reduce the risk of complications ^[9]^.

Despite the substantial increase in the incidence of CD over the past few decades, the impact of early diagnosis on the disease’s clinical course remains debatable. Extensive research has been conducted to identify the risk factors, potential etiologies, and outcomes associated with CD. However, comprehensive information regarding the significance of early diagnosis in preventing CD-related complications is lacking. This study aimed to analyze the initial clinical presentation and the duration of symptoms until a definitive CD diagnosis is made and to correlate these factors with the development of clinical complications.

## Patients and methods

A retrospective review of the medical records of all CD cases from 2000 to 2022 was conducted. The study’s institutional review board granted ethical approval. All the CD participants’ clinical and socioeconomic characteristics and disease outcomes were recorded.

The inclusion criteria comprised patients admitted to the hospital who were over 18 years of age and diagnosed with Crohn’s between 2000 and 2022. The exclusion criteria included patients with ulcerative colitis, pregnant women, and children.

### Definition of diagnostic delay

The time to diagnosis was the delay between the date the patient was asymptomatic and the beginning of symptoms. As in earlier research, a time to diagnosis longer than the 75th percentile was considered a protracted time to diagnosis, or “diagnostic delay” ^[12,13,14,15]^.

### Data collection

Patient data were collected from the hospital’s electronic database, and clinical characteristics of patients with CD were collected, including age, gender, nationality, body mass index (BMI), portal of admission, presence of risk factors, medical history, and family history of IBD. The clinical presentations included abdominal pain, diarrhea, rectal bleeding, fever, weight loss, reduced appetite, fatigue, aphthous ulcers, anemia, and recurrent perineal fistulae. Complications, such as strictures, bowel obstruction, perforations, fistulae, abscesses, anal fissures, ulcers, malignancy, oxalate calculi, sclerosing cholangitis, osteomalacia, osteoporosis, and malnutrition, along with recurrence of these complications, have been documented.

Investigations and laboratory tests included biochemical markers, stool analysis, antibodies, endoscopy, colonoscopy, biopsy, upper gastrointestinal series, MRI, CT tomography scan, complete blood count (CBC), iron levels, vitamin B12 levels, fecal alpha-1 antitrypsin, and fecal calprotectin levels. Additionally, the use of medications such as infliximab, budesonide, prednisone, azathioprine, mesalamine, hydrocortisone, Asacol, dexamethasone, and Pentasa was recorded, including the date of drug initiation and duration of use.

### Ethical considerations

This study received ethical approval from the IRB.

### Data analysis

Data were statistically analyzed using the SPSS application version 26. The Chi-square test (χ2) was applied to the qualitative data expressed as numbers and percentages to investigate the association between the variables. Quantitative data were expressed as mean and standard deviation (mean ± SD), where the Mann–Whitney test was applied for non-parametric variables, and multivariate logistic regression analysis was used to assess factors associated with late CD diagnosis among the studied patients. Odds ratios (OR) were calculated using a 95% confidence interval (CI). Compared to people not exposed to a particular risk, the OR indicates the likelihood that those participants would experience a health issue. Once the off-impacts of other confounders are eliminated, the effects of the risk variables are calculated as independent ORs using regression analysis. Statistical significance was set at a *P* value of <0.05.

## Results

Among the studied 149 patients with CD, the mean age was 35.85 ± 12.54 years, the mean age at diagnosis was 28.43 ± 12.12 years, and the mean BMI was 22.78 ± 6.58 kg/m^2^. The mean disease duration for patients was 7.72 ± 4.02 years, the mean duration from onset of symptoms to the definitive diagnosis was 1.93 ± 3.18 years, and the mean duration from diagnosis to occurrence of complications was 2.18 ± 2.95 years. The results showed that 57% of the patients were males, 80.5% were of Saudi nationality, and 47% were admitted through the ER. Most patients (77.2%) were diagnosed using colonoscopy with biopsy. Moreover, 8.7% had a family history of CD, and 30.9% had either a risk factor or a past medical history. The studied patients, 118 (79.2%), had an early diagnosis, whereas 31 (20.8%) had a late diagnosis. On comparing patients with early CD diagnosis with those with late diagnosis, it was found that patients with a late diagnosis had a significantly longer mean duration from the onset of symptoms to the definitive diagnosis and a longer mean duration from diagnosis to the occurrence of complications (*P* < 0.05). At the same time, patients with a late diagnosis were significantly non-Saudi (35.5% vs 15.3%) (*P* < 0.05). However, a higher percentage of patients with an early diagnosis had a family history of CD (10.2% vs 3.2%) and a higher percentage of patients with risk factors for CD (21.2% vs. 12.9%). No non-significant relationship was found between these two variables (*P* ≥ 0.05) table 1.

**Table 1:** Comparison between patients with early and late CD diagnosis according to their demographic data, clinical data, portal of admission, family history, presence of risk factors, and past medical history (No. 149)

Variable	Variable Type	Type of CD diagnosis		χ2	*P* value
	Total	Early CD diagnosis No. (%)	Late CD diagnosis No. (%)		
Age (years) (Mean ± SD)	35.85 ± 12.54	34.74 ± 11.59	40.06 ± 15.13	1.79*	0.073
Age at diagnosis (years) (Mean ± SD)	28.43 ± 12.12	27.78 ± 11.65	30.9 ± 13.69		
BMI (kg/m^2^) (Mean ± SD)	22.78 ± 6.58	22.38 ± 6.2	24.34 ± 7.82	1.06*	0.288
Disease duration (years) (Mean ± SD)	7.72 ± 4.02	7.34 ± 3.79	9.16 ± 4.57	1.07*	0.284
Duration from onset of symptoms till the definitive diagnosis (years) (Mean ± SD)	1.93 ± 3.18	0.65 ± 0.62	6.79 ± 4.19	8.58*	**<0.001**
Duration from diagnosis till occurrence of complications (years) (Mean ± SD)	2.18 ± 2.95	1.55 ± 2.18	4.51 ± 4.1	4.58*	**<0.001**
Gender					
Female	64 (43)	46 (39)	18 (58.1)	3.64	0.056
Male	85 (57)	72 (61)	13 (41.9)		
Nationality					
Non-S	29 (19.5)	18 (15.3)	11 (35.5)	6.41	**0.011**
S	120 (80.5)	100 (84.7)	20 (64.5)		
Portal of admission					
Daycare unit	4 (2.7)	4 (3.4)	0 (0.0)	4.32	0.364
Endoscopy unit	4 (2.7)	2 (1.7)	2 (6.5)		
ER	70 (47)	53 (44.9)	17 (54.8)		
OPD	73(47.7)	59(50)	12(38.7)		
Method of diagnosis					
Colonoscopy with biopsy	125 (83.9)	100 (84.8)	25 (80.6)	6.58	0.253
CT scan	4 (2.7)	4 (3.4)	0 (0.0)		
Exploratory laparotomy with biopsy	10 (6.7)	6 (5.1)	4 (12.9)		
Laparoscopy with biopsy	2 (1.3)	2 (1.7)	0 (0.0)		
Panendoscopy with biopsy	8 (5.4)	16 (13.6)	3 (9.7)		
Family history					
Positive	13(8.7)	12 (10.2)	1 (3.2)	3.84	0.147
Presence of risk factors					
Yes	46 (30.9)	25 (21.2)	4 (12.9)	1.29	0.523
Past medical history					
Yes	46 (30.9)	34 (28.8)	12 (38.7)	1.21	0.545

*Mann–Whitney test.

The clinical presentations and complications are illustrated in Table 2. The most common clinical presentations were abdominal pain (91.3%), diarrhea (62.4%), anemia (53%), and weight loss (50.3%). The most common complications were fistulas (34.2%), strictures and obstructions (31.5%), malnutrition (29.5%), and abscesses(24.8%). Patients with an early CD diagnosis had a significantly higher prevalence of reduced appetite (44.1% vs. 22.6%) and fatigue (55.1% vs. 25.8%) than patients with a late diagnosis (*P* < 0.05). Although patients with an early CD diagnosis had a higher prevalence of abdominal pain, diarrhea, rectal bleeding, weight loss, aphthous ulcers, anemia, and recurrent perineal fistulae than patients with a late diagnosis, this difference was not significant (*P* ≥ 0.05). Regarding CD complications, patients with a late diagnosis had a significantly higher prevalence of oxalate calculi than those with an early diagnosis (6.5% vs. 0.8%) (*P* < 0.05). Even though patients with a late CD diagnosis had a higher prevalence of abdominal perforation, fistula, abscess, osteomalacia, and osteoporosis than patients with an early diagnosis, this difference was not significant (*P* ≥ 0.05). The same non-significant relationship was found between early and late diagnosis according to the recurrence of complications (*P* ≥ 0.05).

**Table 2:** Comparison between patients with early and late CD diagnosis according to clinical presentations, complications, and recurrence of complications (No. 149)

Variable		Type of CD diagnosis		χ2	*P* value
	Total	Early CD diagnosis No. (%)	Late CD diagnosis No. (%)		
Clinical presentations					
Abdominal pain	136 (91.3)	111 (94.1)	25 (80.6)	5.55	0.018
Diarrhea	93 (62.4)	76 (64.4)	17 (54.8)	0.95	0.328
Rectal bleeding	43 (28.9)	37 (31.4)	6 (19.4)	1.72	0.189
Fever	43 (28.9)	34 (28.8)	9 (29)	0.001	0.981
Weight loss	75 (50.3)	63 (53.4)	12 (38.7)	2.11	0.146
Reduced appetite	59 (39.6)	52 (44.1)	7 (22.6)	4.73	**0.029**
Fatigue	73 (49)	65 (55.1)	8 (25.8)	8.42	**0.004**
Aphthous ulcers	19 (12.8)	18 (15.3)	1 (3.2)	3.19	0.074
Anemia	79 (53)	65 (55.1)	14 (45.2)	0.97	0.325
Recurrent perineal fistulae	17 (11.4)	14 (11.9)	3 (9.7)	0.11	0.733
Complications					
Stricture & obstruction	47 (31.5)	38 (32.2)	9 (29)	0.11	0.735
Perforation	10 (6.7)	6 (5.1)	4 (12.9)	2.39	0.122
Fistula	51 (34.2)	40 (33.9)	11 (35.5)	0.02	0.868
Abscess	37 (24.8)	27 (22.9)	10 (32.3)	1.15	0.282
Anal fissures	18 (12.1)	16 (13.6)	2 (6.5)	1.16	0.28
Ulcers	31 (20.8)	26 (22)	5 (16.1)	0.52	0.471
Malignancy	16 (10.7)	13 (11)	3 (9.7)	0.04	0.83
Oxalate calculi	3 (2)	1 (0.8)	2 (6.5)	3.9	**0.048**
Sclerosing cholangitis	2 (1.3)	2 (1.7)	0 (0.0)	0.53	0.466
Osteomalacia	3 (2)	2 (1.7)	1 (3.2)	0.29	0.589
Osteoporosis	8 (5.4)	6 (5.1)	2 (6.5)	0.09	0.764
Malnutrition	44 (29.5)	38 (32.2)	6 (19.4)	1.94	0.163
Recurrence	56 (37.6)	44 (37.3)	12 (38.7)	3.9	0.142

Most patients (74.5%) used disease-modifying agents, including Prednisone (56.8%), Infliximab (54.1%), Azathioprine (44.1%), and Hydrocortisone (42.3%), the most commonly used. Almost half of the patients (47.7%) started using these drugs after the diagnosis of CD, and the mean duration of use was 2.84 ± 3.13 years. Patients with late CD diagnosis had significantly higher usage of Asacol (4.3% vs. 0%) than those with early diagnosis (*P* < 0.05). Although patients with a late diagnosis had a higher prevalence of Prednisone, Pentasa, or Dexamethasone use, this difference was not significant (*P* ≥ 0.05) (Table 3).

**Table 3:** Comparison between patients with early and late CD diagnosis according to the pattern of disease-modifying agents’ use (No. 149)

Variable		Type of CD diagnosis		χ2	*P* value
	Total	Early CD diagnosis No. (%)	Late CD diagnosis No. (%)		
Did the patient use a disease-modifying agent?					
No	38 (25.5)	30 (25.4)	8 (25.8)	0.002	0.965
Yes	111 (74.5)	88 (74.6)	23 (74.2)		
If yes, which medications were used?					
Infliximab	60 (54.1)	52 (59.1)	9 (39.1)	2.93	0.087
Budesonide	7 (6.3)	6 (6.8)	1 (4.3)	0.18	0.664
Prednisone	63 (56.8)	49 (55.7)	14 (60.7)	0.2	0.655
Azathioprine	49 (44.1)	39 (44.3)	10 (43.5)	0.005	0.942
Mesalamine	30 (27)	22 (25)	8 (24.8)	0.88	0.347
Hydrocortisone	47 (42.3)	39 (44.3)	8 (34.8)	0.67	0.41
Asacol	1 (0.9)	0 (0.0)	1 (4.3)	3.86	**0.049**
Pentasa	4 (3.6)	2 (2.3)	2 (8.7)	2.16	0.141
Dexamethasone	8 (7.2)	6 (6.8)	2 (8.7)	0.09	0.757
Since when did the patient start using these drugs?					
After the diagnosis of CD	71 (47.7)	56 (57.5)	15 (48.4)	0.02	0.989
Before the diagnosis of CD	40 (26.8)	32 (27.1)	8 (25.8)		
None	38 (25.5)	30 (25.4)	8 (25.8)		
For how long did the patient use the disease-modifying agents? (Duration of drug use) (years) (Mean ± SD)	2.84 ± 3.13	2.72 ± 2.94	3.33 ± 3.78	0.56*	0.572

*Mann–Whitney test.

Among the studied patients, the most common biomarkers detected were C-reactive protein (CRP), Erythrocyte Sedimentation Rate (ESR) (87.9%), stool analysis (69.8%), and 26.2% showed Antineutrophil Cytoplasmic Antibodies (ANCA), Anti-Saccharomyces cerevisiae Antibodies (ASCA). A detailed illustration of the investigations and laboratory analyses performed on patients is presented in Table 4. Patients with an early CD diagnosis had a significantly higher percentage of patients with their B12 levels (*P* < 0.05). However, all investigations or other laboratory analyses found a non-significant difference between patients with early and late CD diagnoses (*P* ≥ 0.05).

**Table 4:** Comparison between patients with early and late CD diagnosis according to investigations and laboratory analysis (No. 149)

Variable		Type of CD diagnosis		χ2	*P* value
	Total	Early CD diagnosis No. (%)	Late CD diagnosis No. (%)		
Biochemical markers					
C-reactive protein (CRP)	12 (8.1)	10 (8.5)	2 (6.5)	1.2	0.752
C-reactive protein (CRP), erythrocyte sedimentation rate (ESR)	131 (87.9)	104 (88.1)	27 (87.1)		
No	2 (1.3)	1 (0.8)	1 (3.2)	2.24	0.325
Not mentioned	4 (2.7)	3 (2.5)	1 (3.2)		
Stool analysis	104 (69.8)	85 (72)	19 (61.3)		
Antibodies					
Anti-Saccharomyces cerevisiae antibodies (ASCA)	6 (4)	6 (5.1)	0 (0.0)	5.71	0.456
Antineutrophil cytoplasmic antibodies (ANCA)	11 (7.4)	9 (7.6)	2 (6.5)		
Antineutrophil cytoplasmic antibodies (ANCA), anti-Saccharomyces cerevisiae antibodies (ASCA)	39 (26.2)	31 (26.3)	8 (25.8)		
Antineutrophil cytoplasmic antibodies (ANCA), anti-Saccharomyces cerevisiae antibodies (ASCA), leucine-rich alpha-2 glycoprotein (LRG1)	7 (4.7)	5 (4.2)	2 (6.5)		
Leucine-rich alpha-2 glycoprotein (LRG1), not mentioned	1 (0.7)	0 (0.0)	1 (3.2)		
No	46 (30.9)	36 (30.5)	10 (32.3)		
Not mentioned	39 (26.2)	31 (26.3)	8 (25.8)		
Colonoscopy	202 (135.6)	166 (140.7)	36(116.1)	5.61	0.832
Biopsy	122 (81.9)	96 (81.4)	26 (83.9)	0.32	0.852
Upper GI series	69 (46.3)	54 (45.8)	15 (48.4)	0.31	0.855
MRI	77 (51.7)	64 (45.2)	13 (41.9)	1.87	0.392
CT scan	100 (67.1)	80 (67.8)	20 (64.5)	0.43	0.806
CBC	145 (97.3)	114 (96.6)	31 (100)	1.08	0.583
Iron levels	117 (78.5)	97 (82.2)	20 (64.5)	5.26	0.071
B12 levels	109 (73.2)	91 (77.1)	18 (58.1)	4.53	**0.033**
Fecal alpha 1 antitrypsin (FA)	29 (19.5)	27 (22.9)	2 (6.5)	4.94	0.176
Fecal carprofen	40 (26.8)	36 (30.5)	4 (12.9)	4.63	0.201
Resection	53 (35.6)	41 (34.7)	12 (38.7)	0.4	0.815
Stricturoplasty	6 (4)	5 (4.2)	1 (3.2)	0.06	0.899
Stoma	26 (17.4)	23 (19.5)	3 (9.7)	5.29	0.071
Abscess drainage	30 (20.1)	20 (16.9)	10 (32.3)	3.57	0.059

Multivariate logistic regression analysis assessed the risk factors (independent predictors) of late CD diagnosis among the studied patients. Variables found to be significant in the univariate analysis were included in the regression model. None of these important variables (Nationality, reduced appetite, fatigue as clinical presentations, or the presence of oxalate calculi as a complication) were found to be risk factors for late CD diagnosis among the studied patients (Table 5).

**Table 5:** Multivariate logistic regression analysis of risk factors of late CD diagnosis among studied patients

Variable	B	Wald	*P* value	Odds ratio (CI:95%)
Nationality	0.81	2.84	0.092	2.25 (0.87–5.8)
Reduced appetite in presentation	0.21	0.11	0.732	0.8 (0.24–2.72)
Fatigue in presentation	0.96	2.69	0.1	2.62 (0.82–8.34)
Oxalate calculi as a complication	2.05	2.36	0.12	0.8 (0.56–1.17)

## Discussion

This retrospective study at our hospital assessed the association between early CD diagnosis and the development of clinical complications among 149 patients. The results demonstrated significant differences between patients with early and late diagnoses in terms of the duration from the onset of symptoms until definitive diagnosis and from the onset of diagnosis to the occurrence of complications.

A key finding was that patients with a late diagnosis had significantly longer durations from the onset of symptoms until definitive diagnosis (6.79 years vs. 0.65 years) and from diagnosis until complications (4.51 years vs. 1.55 years). This suggests that patients with a late diagnosis had significantly longer durations from symptom onset to a delayed diagnosis. This may contribute to a higher risk of complications, reinforcing the importance of early detection and intervention in managing CD. The results of this study are consistent with a systematic review published in 2022, which found that identifying “early disease” is the first step in implementing a successful treatment plan. Several studies have discussed the impact of early CD diagnosis on disease outcomes. Most of the information came from anti-TNF drugs as monotherapy or immunosuppressants. The long-term outcomes showed that early immunosuppressants or anti-TNF medications notably decreased the risk of CD-related surgeries. Furthermore, individuals who received treatment early during their illness had reduced hospitalization requirements and disease-related complications ^[16]^. According to a 2023 systematic review, the early diagnosis of CD is crucial for slowing the disease progression and reducing the need for surgical procedures. This study also emphasizes the need to develop better methods for early diagnosis and intervention ^[17]^.

Determining the optimal diagnostic method is crucial for CD management. In this study, most patients were diagnosed by colonoscopy with biopsy. The American College of Gastroenterology Clinical Guideline on the Management of Crohn’s Disease in Adults suggests that colonoscopy with biopsy remains the gold standard for diagnosing CD, which lends credence to this conclusion ^[18]^.

The most common complications noted in patients with a late diagnosis of CD were fistulae and intestinal obstruction, all of which required surgical interventions. Our results are similar to those observed in a prospective cohort study in France, which discussed the correlation between the late diagnosis of CD and the need for surgical interventions in disease management ^[19]^. Results showed no statistical difference was associated with early and late diagnoses regarding patient factors, including age, disease phenotype, need for immunosuppressant therapy, or chemotherapy. However, a higher risk of early surgical interventions resulted from late diagnosis. Furthermore, a recent cohort study conducted on Chinese patients showed that diagnostic delay was associated with a poor outcome of CD, especially with a higher risk of surgery ^[12]^.

Similarly, a retrospective study in Korea concluded that delayed diagnosis was significantly associated with an increased risk of intestinal surgeries in patients with CD ^[20]^. Additionally, a recent systematic review conducted in the UK discovered that a delay in CD diagnosis might result in disease progression and complications such as strictures, fistulae, and the necessity for surgery. Consequently, these studies suggest minimizing diagnostic delays may improve management and results, underscoring the significance of prompt access to specialized care and precise diagnostic techniques ^[17]^.

Most patients in our study used disease-modifying agents such as Prednisone, Infliximab, Azathioprine, Hydrocortisone, and other therapeutic measures. The use of Prednisone, Pentasa, or Dexamethasone in patients with late-stage CD was higher than that in patients with early diagnosis; however, this difference was not statistically significant. According to a recent GETAID research, no optimal therapeutic medical techniques are available due to the lack of established prognostic variables for CD. For example, azathioprine, within 6 months of CD diagnosis, did not prolong the duration of clinical remission any longer than the standard therapy. Some people suffer from a severe illness with a poor prognosis, whereas others have an indolent condition with few flare-ups. Consequently, not every patient requires intensive care during diagnosis ^[12]^. According to a recent study conducted in Italy, Early therapeutic intervention with disease-modifying agents, such as biological drugs, may result in complete disease control and prevention of disease progression, irreversible damage, minimal side effects, and enhanced quality of life. Early treatment may help avoid long-term CD-related damage, as demonstrated by the significantly lower likelihood of perianal surgery in patients receiving azathioprine ^[21]^.

Based on our study results, we hypothesized that a positive family history of CD contributed to the early diagnosis of CD, although the association was not statistically significant. Comparably, an Indian study of 720 patients found that a lack of family history, advanced age at diagnosis, and low educational attainment were among the factors causing delays in CD diagnosis ^[15]^. Furthermore, a prospective cohort study that included 2,136 patients with IBD discovered that individuals with a positive family history typically experienced an earlier onset of CD than those without a family history. As a result, a younger age of diagnosis is likely linked to a positive family history ^[19]^. The study also discovered that non-Saudi patients were more likely to have a delayed diagnosis, suggesting possible socioeconomic variables influencing healthcare utilization or accessibility among various nationalities. Similarly, a Brazilian study of 658 patients raised the possibility that cultural and geographic variables affected the timing of diagnosis ^[22]^. Conversely, a prospective cohort study at a hospital claimed that patient demographics did not impact diagnosis delay ^[2]^.

This study emphasizes raising awareness and implementing prompt diagnostic procedures for CD, especially for individuals with limited access to healthcare facilities.

Enhancing diagnostic methods and ensuring timely intervention may help reduce the severity of CD-related complications. A cohort study conducted in Brazil stated that it is critical to obtain an early diagnosis to initiate treatment for these conditions as soon as possible ^[23]^. Thus, fundamental research on CD emphasizes the significance of prompt diagnosis and the need for comprehensive, multidisciplinary approaches to care.

### Limitations

This study has some limitations, including its retrospective design and single-center setting, which may limit the generalizability of the findings. Future studies should focus on prospective studies with more extensive and diverse populations to validate these results.

## Conclusion

In this study, 20.8% of CD cases were diagnosed late. Patients with a delayed diagnosis were more likely to be non-S. They exhibited a significantly longer duration from symptom onset to definitive diagnosis and from diagnosis to the development of complications. A notable correlation was also observed between Asacol use, increased oxalate calculi prevalence in the kidney, and late diagnosis. However, multivariate logistic regression analysis did not identify specific risk factors for delayed CD diagnosis. These findings highlight the need for improved diagnostic strategies and increased awareness to facilitate early detection and enhance patient outcomes. Further multicenter longitudinal studies are required to clarify the relationship between early diagnosis and the progression of CD-related clinical complications.

## Data Availability

None.

## Research registration unique identifying number (UIN)

Name of the registry: Research Registry. Unique Identifying number or registration ID: research registry 10966. Hyperlink to the registration (must be publicly accessible): https://www.researchregistry.com/browse-the-registry#home/.
